# Functionalization of Ti-40Nb implant material with strontium by reactive sputtering

**DOI:** 10.1186/s40824-017-0104-8

**Published:** 2017-10-10

**Authors:** Markus Göttlicher, Marcus Rohnke, Yannik Moryson, Jürgen Thomas, Joachim Sann, Anja Lode, Matthias Schumacher, Romy Schmidt, Stefan Pilz, Annett Gebert, Thomas Gemming, Jürgen Janek

**Affiliations:** 1 Institute of Physical Chemistry and Center of Materials Research, Justus-Liebig-University of Giessen, Heinrich-Buff-Ring 17, 35392, Giessen, Germany; 2 IFW Dresden, Institute for Complex Materials, Helmholtzstrasse 20, 01069 Dresden, Germany; 3 Centre for Translational Bone, Joint and Soft Tissue Research, Faculty of Medicine and University Hospital, Technische Universität Dresden, Fetscherstrasse 74, 01307 Dresden, Germany

**Keywords:** Surface coating, Titanium alloy, Osteoporosis, Strontium release, Biocompatibility, Plasma deposition

## Abstract

**Background:**

Surface functionalization of orthopedic implants with pharmaceutically active agents is a modern approach to enhance osseointegration in systemically altered bone. A local release of strontium, a verified bone building therapeutic agent, at the fracture site would diminish side effects, which could occur otherwise by oral administration. Strontium surface functionalization of specially designed titanium-niobium (Ti-40Nb) implant alloy would provide an advanced implant system that is mechanically adapted to altered bone with the ability to stimulate bone formation.

**Methods:**

Strontium-containing coatings were prepared by reactive sputtering of strontium chloride (SrCl_2_) in a self-constructed capacitively coupled radio frequency (RF) plasma reactor. Film morphology, structure and composition were investigated by scanning electron microscopy (SEM), time of flight secondary ion mass spectrometry (ToF-SIMS) and X-ray photoelectron spectroscopy (XPS). High-resolution transmission electron microscopy (HR-TEM) was used for the investigation of thickness and growth direction of the product layer. TEM lamellae were prepared using the focused ion beam (FIB) technique. Bioactivity of the surface coatings was tested by cultivation of primary human osteoblasts and subsequent analysis of cell morphology, viability, proliferation and differentiation. The results are correlated with the amount of strontium that is released from the coating in biomedical buffer solution, quantified by inductively coupled plasma mass spectrometry (ICP-MS).

**Results:**

Dense coatings, consisting of SrO_x_Cl_y_, of more than 100 nm thickness and columnar structure, were prepared. TEM images of cross sections clearly show an incoherent but well-structured interface between coating and substrate without any cracks. Sr^2+^ is released from the SrO_x_Cl_y_ coating into physiological solution as proven by ICP-MS analysis. Cell culture studies showed excellent biocompatibility of the functionalized alloy.

**Conclusions:**

Ti-40Nb alloy, a potential orthopedic implant material for osteoporosis patients, could be successfully plasma coated with a dense SrO_x_Cl_y_ film. The material performed well in in vitro tests. Nevertheless, the Sr^2+^ release must be optimized in future work to meet the requirements of an effective drug delivery system.

**Electronic supplementary material:**

The online version of this article (10.1186/s40824-017-0104-8) contains supplementary material, which is available to authorized users.

## Background

Titanium and its alloys are well established biomaterials for the production of load-bearing implants for hard tissue replacement and fracture stabilization. They offer beneficial mechanical properties and develop a stable passivating oxide film that ensures excellent biocompatibility and good corrosion resistance [1]. Despite of their successful use as surgical implants the mechanical and surface properties still need to be improved to meet the specific challenges that orthopedics and traumatology are facing. Key weaknesses of currently used α and α/β-type titanium-based implants are their high stiffness mismatch to bone [2] and their biocompatibility, which can be increased further by surface modification to finally enhance osseointegration [3]. As in the future a continuous rise of population’s life expectancy is expected, more patients will suffer from age-related or post-menopausal osteoporosis or its secondary forms. These bone disorders negatively affect mineral density, fragility [4, 5] and regenerative ability of bone [6]. Therefore, it is an important aim to develop advanced metallic implants that are mechanically adapted to the weakened bone structure and ensure fast bone integration by modified surface characteristics [3].

Beta-type Ti-40Nb alloy is a very promising implant material for the treatment of bone fractures because it exhibits a low elastic modulus combined with moderate strength [7]. Besides that, it has been shown that niobium leads to an outstanding corrosion resistance of the passivating oxide film [8]. While this is a major advantage on the one hand, it makes standard surface treatment protocols for metallic implants ineffective on the other hand, so that alternative methods were exploited [9].

Strontium cations (Sr^2+^) are successfully used for the medical treatment of patients with osteoporosis by oral administration of strontium ranelate. In vitro and in vivo studies showed that strontium ions are able to stimulate new bone formation and inhibit excessive bone resorption [10], which is occurring in osteoporotic bone and caused by a characteristic imbalance between bone-building osteoblasts and bone-resorbing osteoclasts [11]. The success of strontium as an anti-osteoporotic drug suffered a setback in 2014, when the European Medicines Agency recommended a restricted use of strontium ranelate due to its adverse effects, which include an increased risk of serious cardiac disorders [12]. To minimize this risk, a locally administered release of strontium, for example by coating of surgical implants, would be highly beneficial [13]. Numerous studies found an increased biocompatibility and upregulation of osteogenesis related genes in vitro [14–16] and an effectively improved implant osseointegration in vivo*,* in bone-healthy [17, 18] and recently in ovariectomized rats [19]. The spatial dispersion of the therapeutic agent Sr^2+^ in rat bone with time in vivo was recently described by Rohnke et al. [20]. It was shown that the Sr^2+^ release and transport from a biomaterial into bone is a usefull concept, which can be mathematically described with the help of Ficks laws of diffusion.

Methods for preparation of strontium-releasing implant coatings involve hydro-thermal synthesis routes [15–17], Sr-substituted hydroxyapatite coating [21, 22], NaOH treatment followed by strontium acetate treatment [14], electrolytic deposition techniques [23, 24] and non-reactive magnetron sputtering [18, 19]. For optimum medical performance of such coatings it is assumed, that ionic strontium needs to be released continuously over a period of several weeks while the pharmaceutical effect threshold is in the range of 0.10 mM and defined by the serum strontium level of postmenopausal osteoporosis patients that were treated with strontium ranelate [25]. However, an initial burst release of strontium after implantation is also unwanted to reduce the risk of a short-time toxic strontium level [18]. In recent approaches such a regulated strontium release was demonstrated by chelation into folic acid [26] and introduction of topographical structures [27].

In the present study, strontium-containing thin films are deposited on Ti-40Nb substrates by reactive sputtering of strontium chloride in a radio frequency (RF) oxygen discharge. The chemical composition and morphology of films were characterized by scanning electron microscopy (SEM), X-ray photoelectron spectroscopy (XPS), secondary ion mass spectrometry (SIMS) and transmission electron microscopy (TEM). By immersion of the coated substrates in Tris-buffered saline (TBS) the strontium release of the thin films was determined using inductively coupled plasma mass spectrometry (ICP-MS). Finally, the biological response of primary human osteoblasts to these films was evaluated in vitro.

## Methods

### Ti-40Nb sample preparation

A beta-phase Ti-40Nb rod with a diameter of 12 mm was prepared by arc melting and cold crucible casting followed by homogenization treatment, as described in detail in reference [28]. Discs with a thickness of 0.7 mm were prepared by cutting the rod using an IsoMet 5000 linear precision saw (Buehler, Switzerland) equipped with a SiC cut-off wheel (Struers, Denmark). Ti-40Nb discs were ground with bound diamond particles (15 μm) using a Phoenix 4000 grinding and polishing machine (Buehler). The discs were polished with a 9 μm diamond suspension (MetaDi, Buehler) and subsequently fine polished with a 4:1 colloidal SiO_2_ (20 nm MasterMet2, Buehler)/H_2_O_2_ (35% HYPROX®, Evonik, Germany) mixture. Finally, the mirror-polished discs were cleaned in acetone, demineralized water and pure ethanol using an ultrasonic bath for 10 min in succession. The samples were stored in ethanol until strontium deposition was performed.

### Plasma setup

**Fig. 1:**
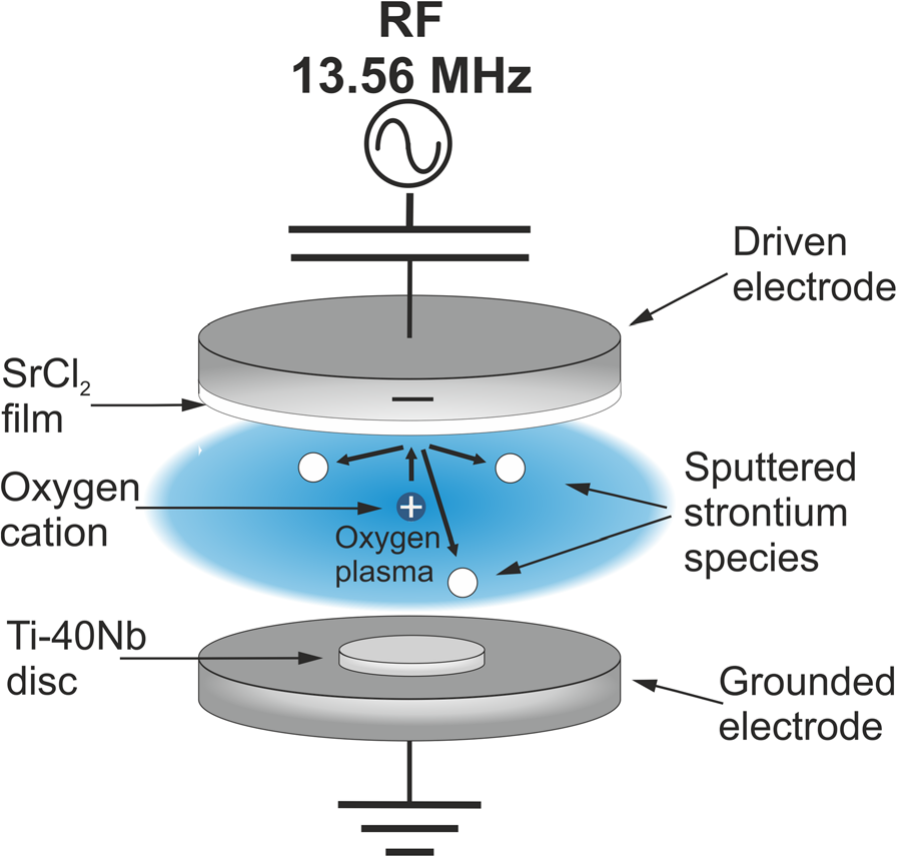
Plasma setup; Schematic sketch of the capacitively coupled RF plasma setup

All deposition experiments were conducted using a self-built plasma setup for excitation of capacitively coupled RF plasma, already introduced in [29] (Fig. 1). Two disc-shaped electrodes of same size were arranged parallel to each other inside a spherical vacuum chamber (approx. 35 cm in diameter), all made of stainless steel. The distance between the electrodes was 3.5 cm, and they are approx. 8.0 cm in diameter. The lower electrode was heatable and attached to the grounded vacuum chamber to ensure adjustment of high self-bias values. The top electrode was powered by an RF generator (PFG RF 300, Trumpf Hüttinger Elektronik, Germany) and matching network system (PFM 1500 A, Trumpf Hüttinger Elektronik), operating at 13.56 MHz.

**Fig. 2:**
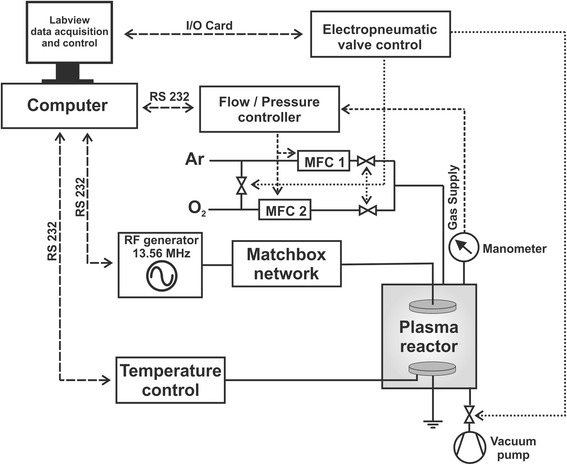
Technical mapping; Technical mapping of the RF plasma equipment

One special feature of the top electrode is a removable lower side that is coated with a SrCl_2_ layer (details to the coating process are described below), therefore acting as strontium-containing target for the sputtering process. For supply of the process gases (Ar, O_2_, both 99.999%), a system of electropneumatic valves (EVI 105 P, Pfeiffer Vacuum, Germany) and mass flow controllers (MKS Instruments, US) was flanged to the vacuum chamber as well as a manometer (Baratron, MKS Instruments, US) to monitor overall gas pressure (Fig. 2).

For evacuation of the vacuum chamber a rotary vane pump (Duo 10, Pfeiffer Vacuum, Germany) was attached. The sample temperature was adjusted using an Eurotherm controller (Model 2416, Schneider Electric, France) and a type K thermocouple (Thermocoax, France) that was located beneath the lower electrode. Electropneumatic valve control, gas flow and pressure control, RF generator and temperature control were connected to a computer to automatize the deposition process using LabVIEW (National Instruments, US) software.

### SrCl_2_ target preparation

A SrCl_2_ layer was prepared onto the removable part of the top electrode (hereinafter referred to as “target electrode”) by dropcasting of a saturated solution of SrCl_2_ (99.9%) and a 1:1 mixture of H_2_O and pure ethanol. The target electrode was placed on a heating plate, fully covered with the solution and subsequently heated to speed up solvent evaporation.

### Strontium functionalization by reactive sputtering

After placing the polished Ti-40Nb samples on the lower, grounded electrode, the plasma chamber was evacuated to a pressure of *p* = 1 Pa several times for complete removal of residual water inside the vacuum chamber including the hygroscopic SrCl_2_ target. Once pumped, the reactor was continuously flooded with a mixture of 0.25 sccm O_2_ and 0.68 sccm Ar resulting in an overall gas pressure of 21 Pa. Strontium deposition was carried out at room temperature with constant RF power of *P* = 100 W and variable dc bias voltage for *t* = 2000 s. Afterwards this procedure was repeated once more. After every deposition process both electrodes were polished with a polishing paper (grain size 4000) and cleaned with acetone to remove passivating oxide films that have been formed due to the oxygen plasma exposure.

### Strontium film characterization

SEM images of the strontium film surface were taken with a MERLIN field emission scanning electron microscope (Carl Zeiss Microscopy, Germany) using an acceleration voltage of 3.0 kV at 4 mm working distance. EDX measurements were carried out with an Oxford Instruments (Abingdon, UK) silicon drift X-Max 50 detector.

For TEM investigations of film morphology (thicknesses, roughness), homogeneity and grain structure thin cross section lamellae were prepared using the focussed ion beam (FIB) technique. A thin slice perpendicular to the sample surface was cut out in a Zeiss Cross Beam 1540 XB (Carl Zeiss Microscopy) with a 30 keV fine focussed gallium ion probe. This lamella was *in situ* transferred and welded on a special copper grid by use of the electron beam under SEM control. The final lamella thickness of less than 100 nm was reached by thinning with a gallium ion probe of lower energy (ca. 5 keV). To reduce the amorphization and the gallium implantation a protection bar of hydrocarbon and hydrocarbon containing platinum was deposited onto the sample surface at the cutting position before starting the procedure. For the TEM analyses we used a TEM/STEM microscope Tecnai F30 ST (FEI Company, US) equipped with Ametek/EDAX (US) windowless SDD for energy dispersive X-ray spectroscopy (EDXS) and Gatan imaging filter 200 for electron energy loss spectroscopy (EELS). The measurements were done using an acceleration voltage of 300 kV reaching a resolution limit of better than 0.2 nm.

The elemental composition of the Sr-coating was analyzed by ToF-SIMS depth profiling using a ToF-SIMS 5-100 machine of IONToF company (Germany). Cation depth profiles were generated by sputtering with 1 keV O_2_^+^ ions in combination with surface analysis using 25 keV Bi^+^ primary ions. The primary ion gun was operated in high current bunched mode with a mass resolution *m*/Δ*m* > 6900 for Ti^+^ (*m*/*z* 47.95). For anion depth profiling 0.5 keV Cs^+^ ions were used. Data were evaluated with SurfaceLab 6.7 software (IONToF company). After depth profiling the sputter craters were measured with a confocal microscope PLu neox 3D (Sensofar Group) to calibrate the sputter time axis. For calibration of the sputter time axis the assumption was made that the sputter yields in the film and in the bulk material are the same.

The surface composition was analyzed by XPS with a PHI Versaprobe 2 instrument (Physical electronics, US) equipped with a monochromatized Al k-alpha source with 1486.6 eV. For depth profiling the instrument is equipped with an Ar^+^ ion sputter gun. Charge neutralization was conducted with ~1 eV electrons and ~10 eV Ar^+^ ions in parallel. The sample was excited with a 200 μm diameter X-ray beam (50 W) and the data was measured with an analyzer pass energy of 23.5 eV and a stepsize of 0.2 eV. For depth profiling, we sputtered with 1 kV beam energy and a raster size of 2 mm × 2 mm for 2 min between each measurement.

### Determination of strontium ion release

For analysis of strontium ion release a 0.05 M Tris-HCl buffer with pH 7.6 containing 0.15 M sodium chloride was prepared by dissolving a TBS BioUltra tablet (Sigma-Aldrich, US) in 500 ml demineralized water. Due to CO_2_ uptake from air the pH decreased slightly after starting the experiment to physiological conditions. Sr functionalized Ti-40Nb samples were prepared and immersed in 5 ml Tris-HCl buffer per sample for one day. For quantitative analysis of the strontium content the buffer solutions were weighted and mixed with concentrated nitric acid at 60 °C. The solutions were diluted with purified water to achieve a suitable concentration range for mass spectrometric analysis. ICP-MS analysis was conducted with an ELEMENT 2 machine from Thermo Fisher Scientific (Germany). Calibration was done by addition of standard Sr^2+^ solutions with 50, 100, 500, 1000 and 5000 ppt. The strontium release experiment was reproduced three times.

### In vitro biological characterization

Biocompatibility of the Sr-functionalized surface was studied in vitro using primary human osteoblasts (hOB). The cells were isolated from spongious bone of human femoral heads derived from two osteoarthritic patients (female, age: 56 and 75 years, moderate to servere state of osteoarthritis) undergoing total hip replacement at the university hospital‚ Carl Gustav Carus‘ Dresden after obtaining informed consent. The ethics commission of Technische Universität Dresden approved the application of hOB for in vitro experiments (no. EK 262092009). The cells were expanded in α-MEM containing 15% fetal calf serum (FCS), 2 mM L-glutamine (L-glu), 100 U/ml penicillin and 100 mg/ml streptomycin (pen/strep) (all from Biochrom, Germany) until passage 3. Sr-functionalized samples (Sr-Ti40Nb) and, as reference, unfunctionalized samples (Ti40Nb) were seeded with 2 × 10^4^ hOB per disc (*d* = 12 mm, *h* = 0.7 mm) and cultivated in α-MEM containing 9% FCS, L-glu, pen/strep as well as 10^−7^ M dexamethasone, 10 mM β-glycerophosphate and 0.05 mM ascorbic acid 2-phosphate (all from Sigma-Aldrich). After 1, 7, 14 and 21 days of cultivation, samples were collected for biochemical and microscopic analysis.

Cell viability and proliferation were assessed by measurement of the intracellular lactate dehydrogenase (LDH) activity and the DNA content; osteogenic differentiation was evaluated by quantification of alkaline phosphatase (ALP) activity. The samples, frozen after cell culture, were thawed and incubated for 50 min in 1% Triton X-100/PBS on ice; cell lysis was supported by 10 min ultrasonication. LDH activity in the lysates was analysed using the CytoTox 96® Non-Radioactive Cytotoxicity Assay (Promega, USA), and the DNA content was determined using the QuantiFluor^®^ Assay (Promega), both according to the manufacturer’s instructions. The measured values were correlated with the cell number using a calibration series. For quantification of the ALP activity, the lysates were incubated with *p*-nitrophenyl phosphate (Sigma-Aldrich) in substrate buffer (0.1 M diethanolamine, 1% Triton X-100, 1 mM MgCl_2_, pH 9.8) at 37 °C for 30 min, followed by absorbance measurement at 405 nm. The amount of *p*-nitrophenolate (pNp) produced by ALP in the cell lysate was calculated using a calibration line. ALP activity was normalized to the cell number to express specific ALP activity (μmol pNp/30 min/10^6^ cells). Cell distribution and morphology on the samples were investigated by fluorescence microscopy: After fixation in 3.7 wt% formaldehyde, actin cytoskeleton and nuclei of the cells were stained by AlexaFluor 488® phalloidin and DAPI (both from Invitrogen, USA), respectively. Fluorescence microscopy was performed using a Keyence BZ-X700 (Keyence Cooperation, Japan).

## Results and discussion

### Strontium oxide chloride film characterization

**Fig. 3:**
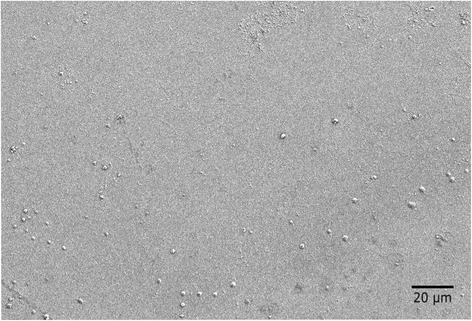
SEM image; Representative scanning electron microscopy image of a coated surface after reactive sputtering of SrCl_2_ in Ar/O_2_ plasma (room temperature *P =* 100 W, 0.25 sccm O_2_, 0.68 sccm Ar, *t* = 2 × 2000 s)

Figure 3 shows a typical SEM image of the film surface after the strontium deposition process. A dense thin film with a very smooth surface was produced whereas additional μm-sized particles were present on the surface. An analysis of the particle composition by EDX (not shown) revealed that they contain strontium, fluorine and chlorine. The origin of this surface contamination is discussed at the end of this section.

**Fig. 4:**
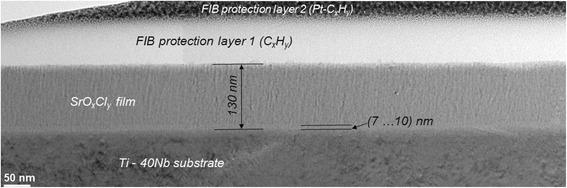
TEM image cross section; Cross section TEM brightfield image of the layer stack showing the thicknesses of the Sr-containing film and interface as well as their crystallinity

**Fig. 5:**
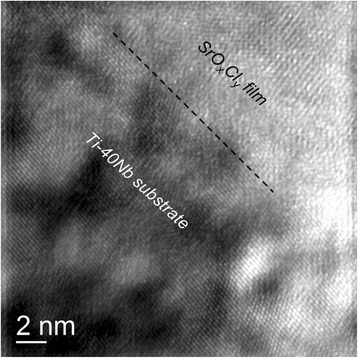
HRTEM image; High resolution TEM image of the interfacial area. The film does not show epitaxial relations to the interface

Figure 4 shows a cross-sectional TEM image of the layer structure after strontium deposition. The thin Sr-containing film, from here termed as SrO_*x*_Cl_*y*_ film, is 130 nm thick including an interlayer of about 10 nm directly above the substrate surface. This interlayer is very likely a passivating Ti-Nb oxide that forms spontaneously after the polishing procedure. The SrO_*x*_Cl_*y*_ film is composed of nanocrystalline grains that are preferably configured in columns with less than 10 nm in width and oriented in growth direction of the layer. The surface roughness of the deposited film is less than 5 nm. A high resolution image of the interface region between substrate and Sr-film in Fig. 5 shows no epitaxial relations between both phases. Although the TEM image reveals a dense and completely adhering coating layer it cannot be excluded that microcracks or spalling occurs, if too much bending is carried out in a convex way.

**Fig. 6:**
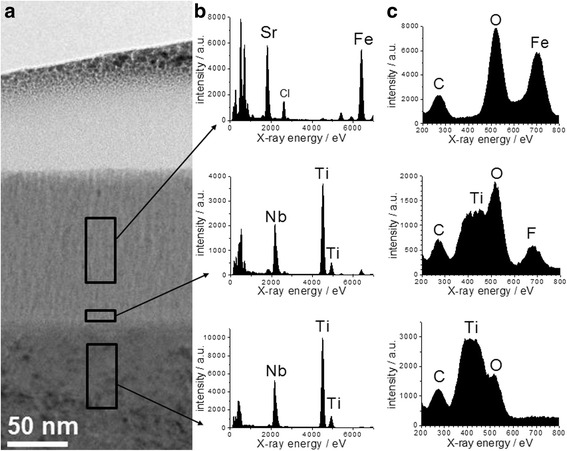
TEM images and EDX spectra; (**a**) TEM brightfield image and EDX spectra acquired from the marked areas (substrate, interface, Sr film). (**b**) X-ray energy range 0 … 7000 eV, (**c**) X-ray energy range 200 … 800 eV

In Fig. 6 EDX spectra of three different regions inside the layer structure are depicted. As expected, titanium and niobium signals emerged in the Ti-40Nb substrate region. At the thin interlayer a higher amount of oxygen was found, compared to the substrate region. This confirms the above mentioned assumption that a passivating oxide film forms an interlayer. A considerable amount of fluorine was found, possibly emerging from condensation of gaseous fluorine compounds inside the vacuum chamber before the deposition process. Potential fluorine sources are Teflon and MACOR® parts inside the vacuum chamber and volatile compounds from the vacuum pump oil or vacuum grease.

Inside the strontium film, mainly strontium, oxygen, chlorine and iron impurities were detected. We conclude that the reaction of SrCl_2_ to SrO in the oxygen discharge is incomplete and that iron was also sputtered from the target electrode, causing this element composition. No fluorine signal was found within the SrO_x_Cl_y_ film so that fluorine incorporation during strontium deposition is relatively low. As the detection limit of TEM-EDXS is 1–2 at.%, this does not exclude smaller fractions of fluorine. As an additional tool for phase characterization we used X-ray diffraction. The obtained data is depicted in the Additional file 1. Unfortunately, the count rate is poor and no well-grounded conclusion is possible. Nevertheless, the small reflexes could assigned to a SrO_*x*_Cl_*y*_ phase.

**Fig. 7:**
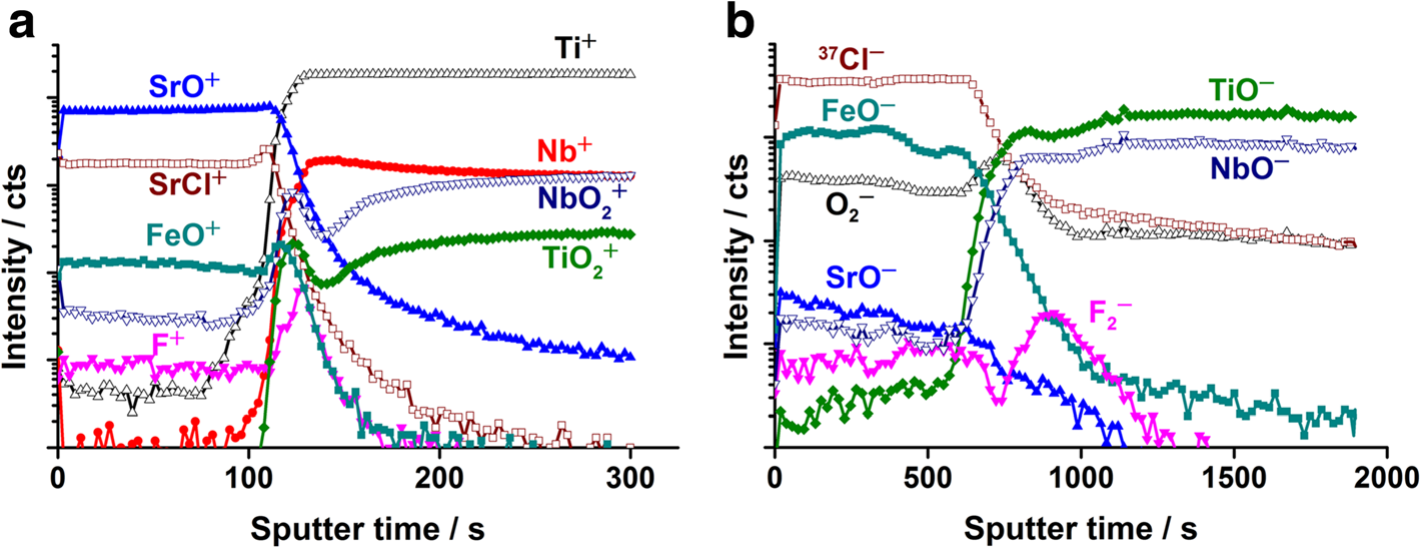
TOF SIMS data; ToF-SIMS depth profiles of the SrO_*x*_Cl_*y*_ coated Ti-40Nb sample. In a) the depth profile obtained from secondary cations is depicted. In b) the depth profile obtained from secondary anions is depicted. SrO_*x*_Cl_*y*_ was deposited at room temperature, *P* = 100 W, 0.25 sccm O_2_, 0.68 sccm Ar, *t* = 5 h

Representative ToF-SIMS anion and cation depth profiles of strontium coated Ti-40Nb are depicted in Fig. 7. As halogens and halogen-containing fragments, such as F^−^, Cl^−^ or SrF^−^ ions are easily ionized, their signal intensity reaches the saturation limit of the secondary ion detector. Therefore, we used specific halogen-containing signals (e.g. ^37^Cl^−^, F_2_^−^) to determine a reliable depth profile of an element species, especially at interfaces. In case of the cations, the Sr^+^ secondary ion signal was oversaturated. From the two depth profiles, we conclude that the deposited strontium film is chemically homogeneous and contains SrO and SrCl_2_ as well as traces of Fe_*x*_O_*y*_. This is in agreement with the reported TEM results.

As mentioned before, F^−^ and SrF^−^ signals in the anion depth profile are oversaturated (not shown in Fig. 7) from the beginning of the spectra. By monitoring the F_2_^−^ and F^+^ signals, we found that the highest fluorine concentration is located at the interlayer. There are also fluorine impurities inside the strontium film, but it is safe to assume that their concentration was below 1 at.%. Besides the fluorine signals also several metal oxide signals (TiO_2_^+^, NbO_2_^+^, O_2_^−^) reach a maximum intensity at the interlayer, which is also in agreement with the TEM results. At higher depth, mainly substrate signals (Ti^+^, Nb^+^) were detected. As observed in [30] the mono-oxygen fragments TiO^−^ and NbO^−^ are characteristic signals in pure Ti-40Nb samples. Due to a high oxygen solubility combined with a high oxygen affinity of the pure metals, these fragments were formed during secondary ion emission.

**Fig. 8:**
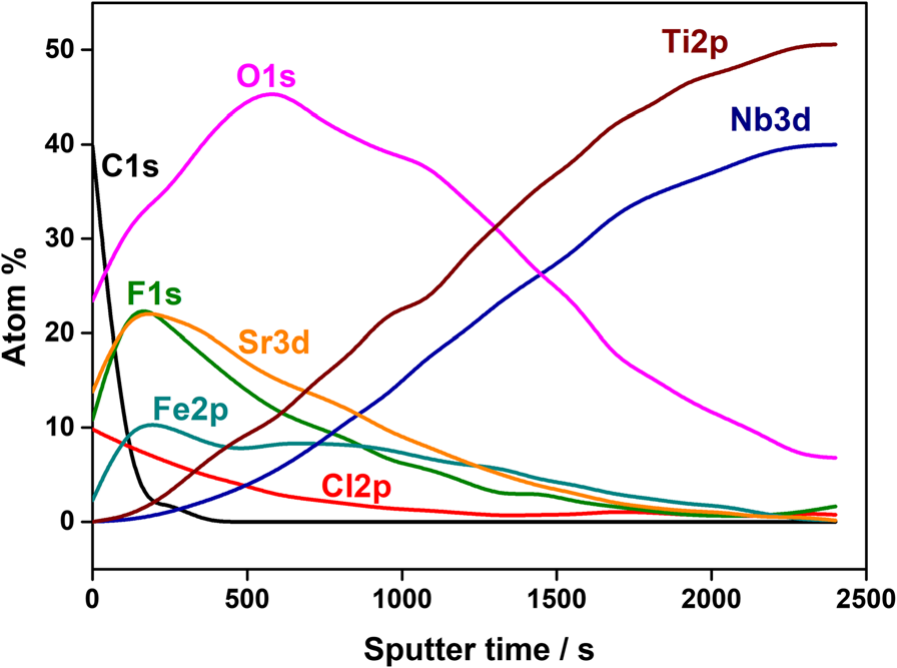
XPS data; XPS depth profile of the SrO_*x*_Cl_*y*_ coated Ti-40Nb sample. SrO_x_Cl_y_ was deposited at room temperature, *P* = 100 W, 0.25 sccm O_2_, 0.68 sccm Ar, *t* = 5 h

XPS measurements were carried out to obtain more accurate chemical information of the coating. In Fig. 8 a sputter depth profile of the SrO_*x*_Cl_*y*_ coated Ti-40Nb sample is shown. We measured detail spectra of C1s, O1s, Cl2p, F1 s, Fe2p, Nb3d, Sr3d and Ti2p. The depth profile supports the findings of the ToF-SIMS and TEM measurements, even though the profile is not as sharp due to a larger analysis spot and a less defined sputter crater. The particles on the surface (Fig. 3) deteriorate the XPS depth resolution. The strontium Sr3d signal is present as SrCO_3_ (only on the surface due to reaction with CO_2_ and water from the lab air). SrF_2_, SrCl_2_ and SrO, correspond well to the respective signals of C1s, O1s, Cl2p and F1 s. The SrF_2_ is a contamination from the thin film deposition process present as small spots on the surface (as shown from SEM/EDXS). Since the XPS spot size is much wider than the SrF_2_ spots the signal superimposed with the SrO_*x*_Cl>_*y*_ film. The film itself consists mainly of SrO and SrCl_2_. Iron is present as another contamination from the deposition process and thus correlates with the strontium signal. The titanium and niobium signals are not present at the surface, indicating a homogeneous film without cracks or pinholes. During depth profiling both the Nb3d as well as the Ti2p signal appear in the fully oxidized state and are reduced to the metal state with ongoing depth profiling. Due to the fitting procedure and background function used, the titanium amount is underestimated. Thus, the stoichiometry at the end of the sputter process is [Ti]/[Nb] = 56/44 instead of the expected atomic ratio of about 74/26. The differing atomic ratio for the investigated alloy in XPS spectra is discussed in detail in [31].

### Strontium ion release

Preliminary studies at different immersion times revealed that strontium is almost completely released into physiological solution after 1 day. By ICP-MS analysis an average Sr^2+^ concentration of 0.05 mM in the buffer solution was quantified. This value is close to the pharmaceutical effect threshold of 0.10 mM for osteoblast activation [10]. In another study Schumacher et al. reported an optimal strontium concentration of 0.01–0.10 mM for stimulation of human-bone-marrow-derived mesenchymal stem cell proliferation and osteogenic differentiation in vitro [32]. However, in contrast to our material, Schumacher et al. tested strontium-modified calcium phosphate bone cements that continuously release 0.03–0.07 mM Sr^2+^ over 20 days. Therefore we conclude that our coating releases enough strontium to stimulate osteoblastic activity, but modifications are required to generate a continuous release. However, in an in vivo environment we have to deal with more complex interactions, e.g. higher strontium concentrations around the implant region because of a lower liquid amount per area that is in contact with the implant. Additionally, adsorbed proteins may block the strontium release. ToF-SIMS analysis of the Ti-40Nb surface after the release experiment revealed only traces of residual strontium. Obviously, the produced coating dissolves quickly, generating an initial burst release of strontium. To produce applicable coatings with optimal strontium release kinetics, it is important that the strontium amount is higher while the coating dissolves with a slower rate and over a longer period. Possible methods to improve the release characteristics of the coating will be the deposition of a less water soluble strontium compound (e.g. strontium phosphate [33]) or the deposition into porous Ti-40Nb implants that might be inserted directly into bone defects [34].

### Biological response of human osteoblasts to SrO_x_Cl_y_-coated Ti-40Nb

**Fig. 9:**
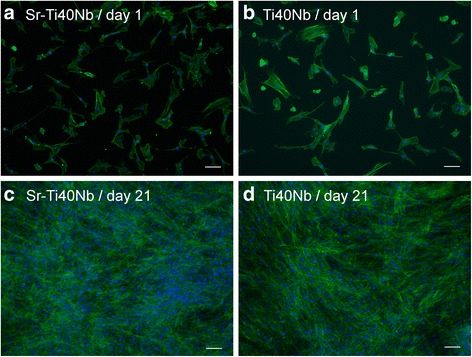
Results cell culture fluorescence micographs; Fluorescence micrographs of hOB cultured on SrO_*x*_Cl_*y*_ coated Ti-40Nb samples (**a**, **c**) and uncoated Ti-40Nb samples (**b**,**d**) for 1 and 21 days, respectively (dual staining of cell nuclei with DAPI (blue) and of actin cytoskeleton with phalloidin (green); scale bar = 100 μm)

**Fig. 10:**
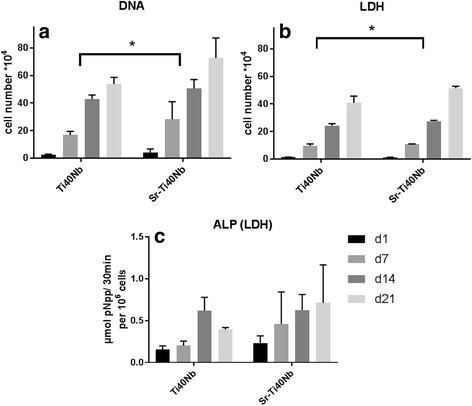
Statistics cell culture data; Cell number increase and osteogenic differentiation of hOB cultured on SrO_*x*_Cl_*y*_-coated (Sr-Ti40Nb) and uncoated (Ti40Nb) Ti-40Nb samples over 21 days. (**a**) DNA content correlated with the cell number, (**b**) intracellular LDH activity correlated with the cell number, (**c**) specific ALP activity. The cell experiment was performed using triplicates; 2-way analysis of variance (ANOVA) was used to evaluate statistical significance at a level of *p* < 0.05. Post-hoc analysis using the Tukey-method was used for multiple comparisons (GraphPad PRISM 7.02)

For probing the biological response human osteoblasts (sustained from osteoarthritis patients) were cultivated on the surface of SrO_*x*_Cl_*y*_-coated Ti-40Nb (Sr-Ti40Nb in Fig. 9) and unmodified Ti-40Nb samples (Ti40Nb in Fig. 9). On both surfaces, the cells showed the typical spreading and cytoskeletal organization 1 day after seeding and their number increased strongly during further cultivation (Fig. 9). Both, measurements of the LDH activity (related to the number of viable cells) and the DNA content (reflecting the total cell number) demonstrated an increase of the cell number over time, which was significantly higher on the strontium-modified samples in the first experiment with hOB from donor 1 (Fig. 10). However, in a second experiment with cells from donor 2, no significant differences were determined between Sr-Ti40Nb and Ti40Nb (data not shown). The activity of the osteoblastic marker ALP increased during the cultivation period of 21 days on both surfaces. However, no significant difference between Sr-Ti40Nb and Ti40Nb was observed (Fig. 10).

The results show excellent biocompatibility of the coating. Because of initial burst release no continuous Sr^2+^ delivery took place and the effect of Sr^2+^ is low because of medium change during cultivation and only minor Sr^2+^ release after medium change.

## Conclusions

Ti-40Nb alloys were successfully plasma coated with a strontium compound. TEM images of cross sections, which were prepared by focused ion beam technique, reveal dense coating layers of more than 100 nm thickness. From the results of the applied surface analytical methods XPS and ToF-SIMS we conclude that the deposited layer is a SrO_*x*_Cl_*y*_ compound. Additionally, traces of F and Fe were found as contaminations from the plasma setup. These contaminations can be avoided in commercial reactors by the use of a special design. Here larger distances between reactor walls and discharge setup as well as better shielding of the built-in components can be realised and should avoid cross contamination with materials sputtered from the setup itself. In vitro release experiments of the coated alloys in aqueous saline solutions reveal that Sr^2+^ is released from SrO_*x*_Cl_*y*_ films too fast. However, we achieved concentrations, that are above the pharmaceutical effect threshold. In cell culture experiments with human osteoblasts a positive effect of the coating on cell proliferation and osteogenic differentiation was observed. Nevertheless, future work will focus on the preparation of thicker coatings with slower Sr^2+^ release by the use of other strontium compounds with lower solubility.

## Additional file

Additional file 1: X-ray diffraction data of surface coating. (PDF 134 kb)

## Abbreviations

ALP: Alkaline phosphatase
ANOVA: Analysis of variance;
DAPI: 4′,6-diamidino-2-phenylindole
DNA: Deoxyribonucleic acid
EDXS: Energy dispersive X-ray spectroscopy
EELS: Electron energy loss spectroscopy
FCS: fetal calf serum;
FIB: Focused ion beam;
hOB: Primary human osteoblasts
HR-TEM: High resolution transmission electron microscopy
ICP-MS: Inductively coupled plasma mass spectrometry
LDH: Lactate dehydrogenase
L-glu: L-glutamine
MFC: Mass flow controller
pen/strep: 100 U/ml penicillin and 100 mg/ml streptomycin
pNp: p-nitrophenolate
RF: Radio frequency
SDD: Silicon Drift detector
SEM: Scanning electron microscopy
STEM: Scanning transmission electron microscopy
TBS: Tris-buffered saline
Ti-40Nb: Titanium-40(weight-%)niobium
ToF-SIMS: Time of flight secondary ion mass spectrometry
XPS: X-ray photoelectron spectroscopy
X-ray: X-radiation
α-MEM: Alpha minimum essential medium
