# Supplementary material for: Functionalization of Ti-40Nb implant material with strontium by reactive sputtering
Source: Biomater Res. 2017 Oct 10;21:18. doi: 10.1186/s40824-017-0104-8 (PMC5634847; doi:10.1186/s40824-017-0104-8)
Supplement: Additional file 1: — X-ray diffraction data of surface coating. (PDF 134 kb) [file 40824_2017_104_MOESM1_ESM.pdf]

Functionalization of Ti-40Nb Implant Material with Strontium by Reactive Sputtering

Markus Goettlicher[1], Marcus Rohnke[1], Yannik Moryson[1], Jürgen Thomas[2], Joachim Sann[1], Anja Lode[3], Matthias Schumacher[3], Romy Schmidt[2], Stefan Pilz[2], Annett Gebert[2], Thomas Gemming[2], Jürgen Janek[1]

[1] Institute of Physical Chemistry & Center of Materials Research, Justus-Liebig-University of Giessen, Heinrich-Buff-Ring 17, 35392 Giessen, Germany

[2] IFW Dresden, Institute for Complex Materials, Helmholtzstrasse 20, 01069 Dresden, Germany

[3] Centre for Translational Bone, Joint and Soft Tissue Research, Faculty of Medicine and University Hospital, Technische Universität Dresden, Fetscherstrasse 74, 01307 Dresden, Germany

X-ray diffraction pattern of surface coating

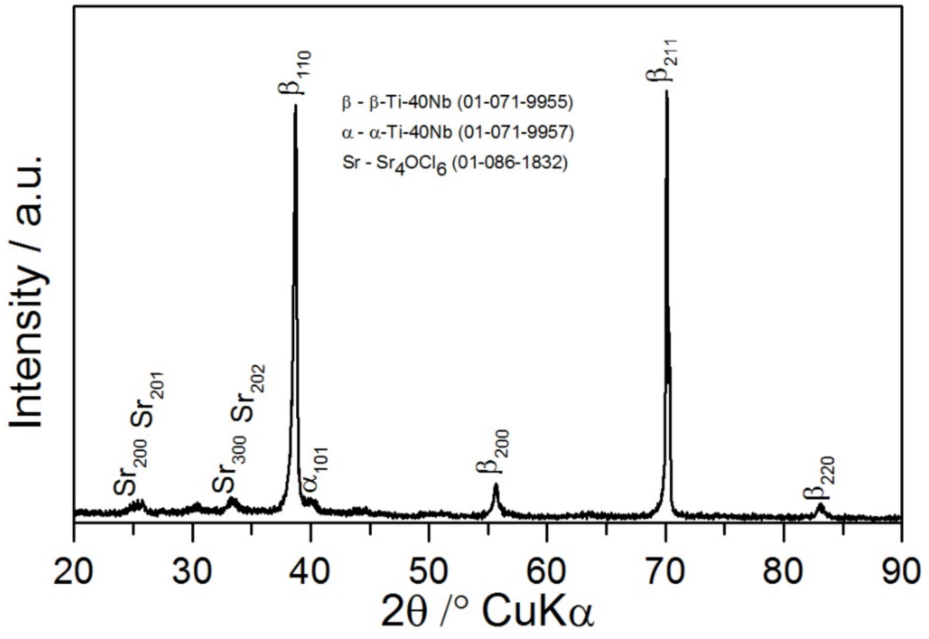

Fig. 1: Gracing incidence diffraction pattern of the deposited layer, incidence angle 2°, in brackets: number of the PDF (powder diffraction file) retrieved from the ICSD database.
